# External quality assessment for molecular detection of sand fly-borne phleboviruses circulating in the Mediterranean Basin

**DOI:** 10.1186/s13071-025-06785-0

**Published:** 2025-05-12

**Authors:** Nazli Ayhan, Cecile Baronti, Laurence Thrion, Gioia Bongiorno, Carla Maia, Remi N. Charrel, CLIMOS WP2 Phlebovirus Working Group, Ozge Erisoz Kasap, Ceylan Polat, Gizem Oguz Kaskan, Bulent Alten, Eduardo Berriatua, José Risueño, Clara Muñoz-Hernández, Edwin Kniha, Katharina Platzgummer, Julia Walochnik, Ricardo Parreira, Yasemin Coşgun, Umut Berberoğlu, Oscar David Kirstein, Victoria Indenbaum, Ravit Koren, Osnat Halpern, Yaniv Lustig, Muhammed Nalcaci, Kardelen Yetismis, Seray Toz, Yusuf Ozbel, Jerome Depaquit, Nalia Mekarnia, Claudia Fortuna, Giulia Marsili

**Affiliations:** 1 Unite des Virus Emergents (UVE), L Université d Aix-Marseille–Universita di Corsica, IRD 190, Inserm 1207–L Institut de recherche biomédicale des armées (IRBA), 19–21, Bd. Jean Moulin, 13005 Marseille, France; 2 Centre National de Reference des Arbovirus, Inserm-IRBA, Marseille, France; 3 Department of Infectious Diseases, Vector-borne Diseases Unit, Istituto Superiore di Sanità, Rome, Italy; 4 Global Health and Tropical Medicine (GHTM), Associate Laboratory in Translation and Innovation Towards Global Health, LA-REAL, Instituto de Higiene e Medicina Tropical (IHMT), Universidade NOVA de Lisboa, Lisboa, Portugal; 5 Laboratoire des Infections Virales Aigues et Tropicales, AP-HM Hopitaux Universitaires de Marseille, Marseille, France

**Keywords:** *Phenuiviridae*, *Phlebovirus*, Toscana virus, Sandfly fever Sicilian virus, External Quality Assessment, *Phlebovirus toscanaense*, *Phlebovirus siciliaense*, PCR, Detection

## Abstract

**Background:**

Sand fly-borne phleboviruses (SbPV) are globally distributed and pose potential public health risks. Despite increased detection in recent decades, detailed knowledge of their ecology, characteristics and clinical relevance remains limited. Many cases of SbPV infection likely go unreported or misdiagnosed due to limited awareness and the lack of standardized screening. The External Quality Assessment (EQA) reported herein was organized within the framework of the European Union CLIMOS (EU Climate Monitoring and Decision Support Framework for Sand Fly-borne Diseases Detection and Mitigation) project. The aim of this EQA was to standardize the detection of phleboviruses in order to provide comparable data to feed mathematical models for the surveillance of the impact of climate changes and environmental parameters on the kinetics and diversity of sand fly species and on sand fly-borne microorganisms.

**Methods:**

Nine laboratories from seven countries participated in the EQA. Each laboratory was provided with eight vials, each containing an anonymous sample; two vials of lyophilized primers and probes to be used for the detection of Toscana virus (TOSV) and several Sandfly fever Sicilian virus (SFSV) species with a reverse-transcriptase PCR (RT-PCR) assay; and one vial of lyophilized primers for the detection of generic phleboviruses with a RT-PCR assay along with the standard operating procedure. The laboratories were instructed to submit their results together with details on the techniques employed.

**Results:**

All nine laboratories successfully detected the two TOSV- and the one SFSV-positive samples. Only one laboratory, using a generic phlebovirus assay, detected all of the targeted phleboviruses.

**Conclusions:**

All participating laboratories successfully identified the two TOSV and one SFSV using the proposed RT-qPCR assays, albeit with some variations in cycle threshold values across laboratories. The detection rate of SbPV was lower with the generic Phlebovirus assay than with the specific real-time RT-qPCR assays. This EQA aimed to assess the SbPV detection capabilities of molecular tools and strengthen their use, thereby supporting the involvement of laboratories in virus discovery and surveillance beyond their core expertise.

**Supplementary Information:**

The online version contains supplementary material available at 10.1186/s13071-025-06785-0.

## Background

Sand fly-borne phleboviruses (SbPV, *Phenuiviridae* family) have a worldwide geographical distribution and represent potential public health concerns [1, 2]. Although the number of recognized SbPV has increased significantly over the two last decades, reflecting heightened surveillance efforts, comprehensive data on the characteristics, ecological cycles and clinical significance of most SbPV remain scarce. Many SbPV continue to be underreported or wrongly identified due to a lack of widespread awareness and systematic screening protocols.

 Toscana virus (TOSV; *Phlebovirus toscanaense*) and Sandfly fever Sicilian virus (SFSV; *Phlebovirus siciliaense*), both human pathogens, are among the most well-characterized SbPV. TOSV is of particular importance due to its ability to cause a spectrum of febrile illnesses, ranging from mild flu-like symptoms, such as fever, headache, fatigue and retro-orbital pain, to more severe neuroinvasive diseases, including meningitis and encephalitis. SFSV, on the other hand, is primarily associated with sandfly fever, commonly referred to as "three-day fever," characterized by a sudden onset of high fever, severe lethargy and general malaise lasting approximately 3–4 days [1–3]. Despite their clinical significance and well-documented history, both of these viruses remain neglected in routine diagnostic testing in many regions, leading to underestimation of their true prevalence and public health impact.

One of the major challenges in SbPV research and diagnosis is the lack of standardized and widely accessible diagnostic tools. While specific assays for TOSV have been developed, they are not routinely implemented in clinical or laboratory settings in many endemic countries, further contributing to their underdiagnosis. Additionally, there is currently no commercially available pan-generic assay capable of detecting all SbPV, despite several individual and comparative diagnostic approaches having been described in recent studies [4]. The absence of such broad-spectrum diagnostic tools hinders comprehensive surveillance efforts, limiting the ability of such efforts to assess the full epidemiological impact of SbPV and their potential to cause emerging infectious diseases.

Vector surveillance programs also play a crucial role in virus screening. Compared to mosquito vectors, sand flies remain largely neglected despite their significance in virus transmission. Studying SbPV in vectors provides valuable insights into vector species specificity and natural transmission cycles, which are essential for understanding virus ecology, evolution and characterization. *Phlebotomus perniciosus* and *Ph. perfiliewi* are recognized as the primary vectors of TOSV and *Ph. papataci* is the primary vector of SFSV. Additionally, recent vector surveillance and virus screening efforts have led to the identification of suggested larger varieties of sand fly vector species [5].

Given the increasing recognition of these viruses and their potential for causing human diseases, it is imperative to enhance surveillance, to improve diagnostic capabilities, to understand the natural cycle and to raise awareness among healthcare professionals and researchers. As a part of the European Union CLIMOS (EU Climate Monitoring and Decision Support Framework for Sand Fly-borne Diseases Detection and Mitigation) project, which aims to mitigate climate change impacts on vector-borne and zoonotic diseases by applying Eco-health and One Health approaches, with a focus on how environmental factors influence sand flies and the spread of sand fly-borne diseases in Europe, we conducted an External Quality Assessment (EQA) with nine laboratories to evaluate and improve detection capacities. This initiative aims to address existing gaps in SbPV research, thereby enabling a more comprehensive understanding of the transmission dynamics and public health implications of these viruses, ultimately contributing to more effective prevention and control strategies. Furthermore, within the framework of the EU CLIMOS project, the generation of comparable data is crucial for feeding the mathematical models that assess the impact of climate change and environmental factors on the dynamics and diversity of sand fly species, as well as on the microorganisms they carry.

The EQA study was designed to address the specific molecular detection of TOSV and several SFSV species, and for generic SbPV identification, using published reverse transcription polymerase chain reaction (RT-PCR) assays. Accordingly, ad-hoc lyophilized primers and probes (Lyo-P&P) were distributed to nine laboratories together with the standard operating procedures (SOP) and inactivated viruses and mock material. Here we report the results and analyze the impact of the RNA purification methods and of the molecular generic kits used for SbPV detection.

## Methods

### External quality assessment

**Fig. 1:**
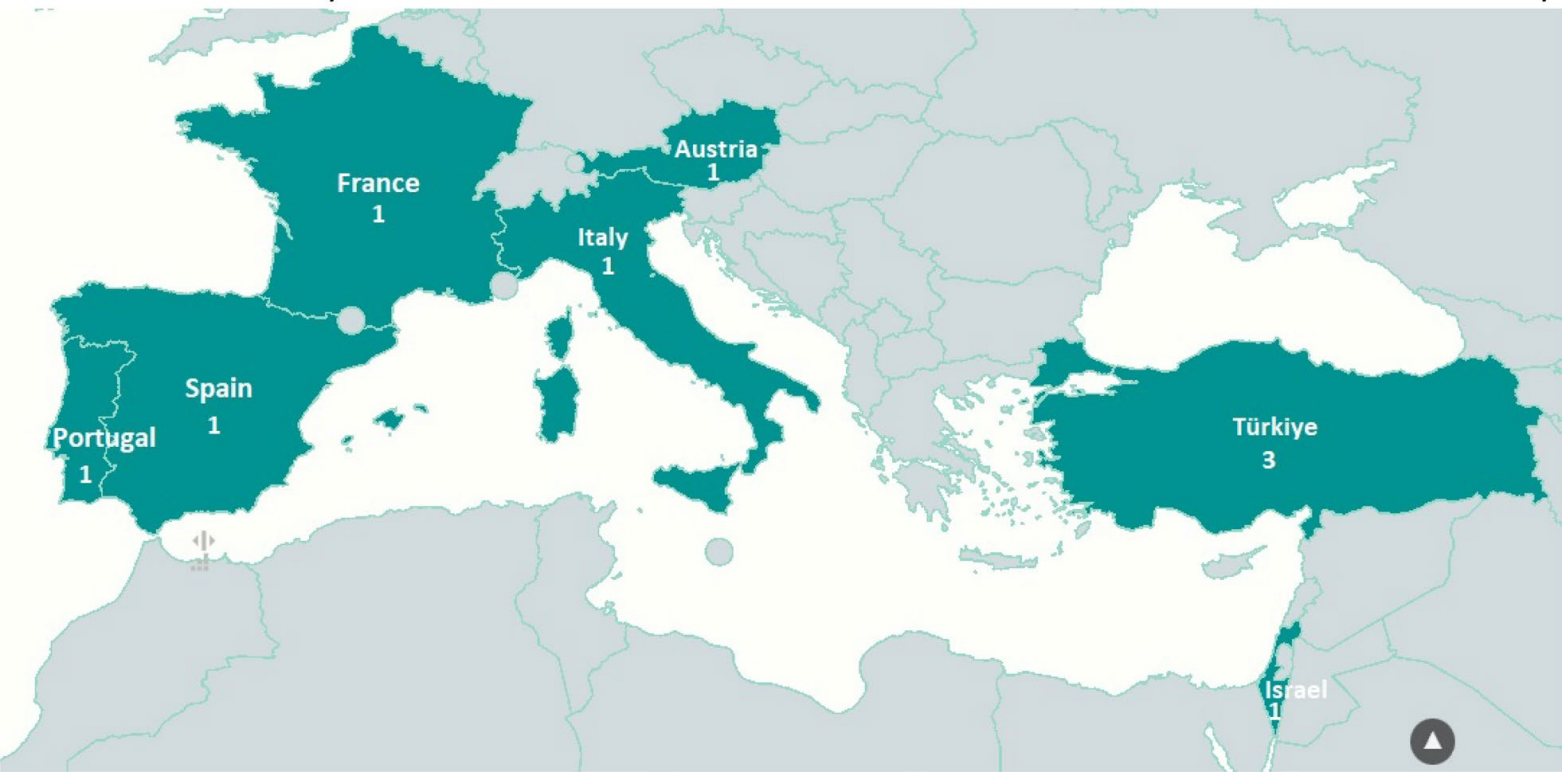
Geographical representation of the nine laboratories from seven countries that participated in the External Quality Assessment. The numbers indicate the number of laboratories in each country

The detection capabilities of nine laboratories from seven countries participating in the EQA, including five laboratories from European/European Economic Area countries (Austria, France, Italy, Portugal and Spain), three laboratories from Türkiye and one laboratory from Israel, were assessed and evaluated by the French reference laboratory (Aix Marseille University, France) between March and September 2023. Each laboratory received a questionnaire for describing the nucleic acid purification method, PCR apparatus and PCR master mix used. After questionnaire completion, each laboratory received eight vials of inactivated virus or mock samples as well as three vials of lyophilized primers and probes with a SOP (Additional file 1: Data 1) for sample manipulation and PCR methodology (Fig. 1).

### Panel composition

#### Sample preparation

The EQA panel consisted of eight anonymized samples including six inactivated virus mentioned in Table 1 and two mock materials (registered non-therapeutic human plasma) (Table 1).

**Table 1:** List of selected phleboviruses included in the sand fly-borne phleboviruses External Quality Assessment panel

Identification number	Virus species	Virus name/abbreviation	Virus strain	EVA number^a^	GenBank number
1	*Phlebovirus salehabadense*	Arbia virus/ARBV	UVE/ARBV/UNK/IT/Phl.35 M6	001v-EVA102	EU266620
2	*Phlebovirus massiliaense*	Massilia virus/MASV	UVE/MASV/2009/FR/M43	001 V-02369	NA
3	*Phlebovirus puniquense*	Punique virus/PUNV	Tunisie2009 T101	001 V-02383	OM362898
4	*Phlebovirus siciliaense*	Sandfly fever Sicilian virus/SFSV	UVE/SFSV/1943/IT/Sabin	001v-EVA7	EF095551
5	*Phlebovirus toscanaense*	Toscana virus/TOSV	UVE/TOSV/2010/TN/ T152, Lineage A	001 V-02119	JX867534, JX867535, JX867536
6	*Phlebovirus toscanaense*	Toscana virus/TOSV	UVE/TOSV/2010/FR/4319, Lineage B	001 V-02442	KC776214 KC776215 KC776216

*NA:* Not available

^a^European Virus Archive (EVA); accessible at https://www.european-virus-archive.com/evag-portal

Non-infectious virus samples were prepared by using virus culture supernatant that was heat-inactivated at 60 °C for 1 h. Inactivation was demonstrated by the absence of cytopathic effect after two passages in Vero cells (ATCC CCL-81; American Type Culture Collection, Manassas, VA, USA) (CVCL_0059) and the lack of increased viral RNA titer in the supernatant 7 days after inoculation as measured by virus-specific RT-PCR assays. Each 2-ml glass vial was filled with 0.4 ml of inactivated material before lyophilization.

#### Lyophilization

##### Preparation and aliquoting

Sucrose was added to the inactivated virus samples to achieve a final concentration of 0.2 M. Tubes were placed on aluminum plates and pre-cooled at − 80 °C. Aliquots of 400 µL per tube were prepared, sealed with caps, and transferred to a pre-frozen lyophilizer (Cryotec—Bench-Top Pilot Lyophilizer).

##### Lyophilization process

The lyophilization cycle was initiated using the Cryotec system. Upon completion, the vacuum was broken by adjusting the vacuum break knob. The chamber was filled with nitrogen by opening the nitrogen valve, and the tubes were sealed under nitrogen conditions to ensure sample integrity.

##### Lyophilized primers and probes (Lyo-P&P)

Primers and probes for two real-time, reverse-transcriptase PCR (RT-qPCR) specific assays for TOSV and Pan-SFSV and primers for one conventional end-point Pan-Phlebovirus RT-PCR assay were lyophilized as described [11] (Table 2). The TOSV trio RT-qPCR assay included, in the same reaction, three monoplex assays [6-8] as previously described [12]. The Pan-SFSV RT-qPCR assay was not specific for SFSV but could also detect other SFSV-like viruses such as Corfou (CFUV), Dashli (DASV) and Toros (TORV) viruses. The Pan-Phlebovirus RT-PCR assay, is one of the most comprehensive assay for detecting phleboviruses [4].

**Table 2:** References, primer sequences, target segments and genes, positions, and amplicon size details of assays used in the External Quality Assessment

Assay	Reference	Primer/Probe	5’ → 3’ Sequence	Target	Position	Amplicon size (in nucleotides)
Toscana virus (TOSV) trio	[6]	STOS-F	TGCTTTTCTTGATGAGTCTGCAG	S RNA, N gene	1718–1807	89
		STOS-R	CAATGCGCTTYGGRTCAAA			
		STOS-P	FAM-ATCAATGCATGGGTRAATGAGTTTGCTTACC-TAMRA			
	[7]	TOS F	GGGTGCATCATGGCTCTT	S RNA, N gene	1381–1531	150
		TOS R	GCAGRGACACCATCACTCTGTC			
		TOS P	FAM-CAATGGCATCCATAGTGGTCCCAGA-TAMRA			
	[8]	TOS-IMT-F	TCTCCCAGGAAATGACATCC	S RNA, N gene	621–725	104
		TOS-IMT-R	AGATGGGWGTCTCTGGTCAT			
		TOS-IMT-P	FAM-TGTGGTYCAAGCAGCACGGGTG-TAMRA			
Pan-SFSV (Sandfly fever Sicilian virus)	[9]	SFSV-All-F	ATGGASGASTACCAGAARATYGC	S RNA, N gene	1655–1761	106
		Corfou-Toros-F	ATGGAGGACTACCAGAAGATCGC			
		Corfou-Toros-R	CTAGCATCAAAACCYTGGTAAGCAAA			
		SFSV-DAHV-R	CTGGCATCAAAYCCYTGATAIGCAAA			
		SFSV-DAHV-F	ATGGACGAGTACCAGAAAATTGC			
		Corfou-Toros-P	FAM-TTCGGTGAGCAGGCTATAGATGA-TAMRA			
		SFSV-P1	FAM-TTTGGAGAACAGGCCATTGATGAG-TAMRA			
		SFSV-P2	FAM-TTTGGAGAGCAGGCTATTGATGAG-TAMRA			
Pan-Phlebovirus	[10]	TBPVL2759F	CAGCATGGIGGICTIAGAGAGAT	L RNA RdRp gene	2786–3300 2786-3309	514
		TBPVL3267R	TGIAGIATSCCYTGCATCAT			
		HRT-GL2759F	CAGCATGGIGGIYTIAGRGAAATYTATGT			523
		HRT-GL3276R	GAWGTRWARTGCAGGATICCYTGCATCAT			

*F* Forward primer, *L* large, *N* viral nucleocapsid protein, *P* probe, *R* reverse primer, *S* small,

### Submission, evaluation and EQA scoring of EQA results

Participants received a Microsoft Excel (Microsoft Corp., Redmond, WA, USA) form to submit EQA results (Additional file 2: Data 2) on which they reported positive/negative results for the three molecular assays and the amplification threshold cycle (Ct) values for the RT-qPCR assays (TOSV trio and Pan-SFSV). Data were collected and analyzed using Microsoft Excel 2011 to calculate the percentage of correct results for each assay and overall.

## Results

### EQA participation

The results of the nine laboratories from seven countries were analyzed, and the results are shown in Table 3.

**Table 3:** Details on RNA extraction, PCR methods and kits and PCR volumes used by laboratories participating in the External Quality Assessment

Laboratory idenification number	Volume used for RNA purification (µl)	Elution volume (µl)	Volume of RNA per RT-PCR (µl)	Kit/apparatus used for extraction	Kit for real-time RT-qPCR	Kit for conventional RT-PCR
Reference Laboratory	200	60	5	Qiagen: EZ1 Advanced XL with virus minikit v2.0 (9018708) (automated)	Thermo Fisher Scientific: RT-qPCRone-step SuperScript III Platinum (11732088)	Thermo Fisher Scientific:SuperScript™ III One-Step RT-PCR System with Platinum (10928042)
1	200	60	5	Resnova: MAGPURIX VIRAL/Pathogen Nucleic Acids Extraction Kit A (ZP02011) (automated)	Meridian Bioscience: 2 × SensiFAST Probe No-ROX Mix Bioline (BIO-76001)	Meridian Bioscience: MyTaqMix One Step RT-PCR Bioline (MDX062)
2	300	50	5	Promega: Maxwell® 16 Viral Total Nucleic Acid Purification Kit (AS1150) (automated)	Promega: GoTaq® Probe 1-Step RT-qPCR System (A6120)	Promega: Access RT-PCR System (A1260)
3	200	60	5	Thermo Fisher Scientific: MagMAX™ Viral/Pathogen Ultra Nucleic Acid extraction kit (A42356) (manual)	Thermo Fisher Scientific: RT-qPCRone-step SuperScript III Platinum (11732088)	Thermo Fisher Scientific: SuperScript™ III One-Step RT-PCR System with Platinum (12574018)
4	400	100	5	Precision System Science Co.: MagLEAD 12gC (MagDEA Dx SV 400) (automated)	Meridian Bioscience: Inhibitor-Tolerant RT-qPCR Mix (MDX016)	Qiagen: OneStep RT-PCR Kit (210212)
5	140	60	5	Qiagen: QiaAmp Viral RNA Mini Lit (52904) (manual)	NZY Tech: NZYSpeedy One-step RT-qPCR Probe Master Mix (MB35201) Thermo Fisher Scientific: Taqman master mix (4444434)	Thermo Fisher Scientific: SuperScript™ IV First-Strand Synthesis System (18091050) Thermo Fisher Scientific: DreamTaq PCR Master Mix (2X) (K1071)
6	200	30	5 in RT-PCR, 4 in qPCR	Qiagen: RNeasy Mini Kit (74104) (manual)	New England Biolabs: Luna Universal Probe One-Step RT-qPCR Kit (E3006S)	Thermo Fisher Scientific: SuperScript™ III One-Step RT-PCR System with Platinum (10928042)
7	200	30	5	Qiagen: RNeasy Mini Kit (74104) (manual)	Thermo Fisher Scientific: RT-qPCRone-step SuperScript III Platinum (11732088)	Thermo Fisher Scientific: SuperScript™ III One-Step RT-PCR (11732020)
8	200	60	5	Qiagen: EZ1 Advanced XL with virus minikit v2.0 (9018708) (automated)	Thermo Fisher Scientific: RT-qPCRone-step SuperScript III Platinum (11732088)	Thermo Fisher Scientific: SuperScript™ III One-Step RT-PCR (11732020)
9	200	50	5	Qiagen: RNeasy Mini Kit (74104) (manual)	Thermo Fisher Scientific: RT-qPCRone-step SuperScript III Platinum (11732088)	Thermo Fisher Scientific: SuperScript™ III One-Step RT-PCR System with Platinum (12574018)

*qPCR* Quantitative PCR,* RT-PCR* reverse-transcriptase PCR,* RT-qPCR* reverse-transcriptase quantitative PCR

### Nucleic acid extraction

The techniques used for nucleic acid extraction are presented in Table 3. Five laboratories used nucleic acid extraction kits manufactured by Qiagen (Hilden, Germany), of which four were manual methods. The other laboratories used the extraction kits manufactured by Resnova s.r.l. (Brescia, Italy) (*n* = 1), Promega (Madison, WI, USA) (*n* = 1), Thermo Fisher Scientific (Waltham, MA, USA) (*n* = 1) and Precision System Science Co., Ltd (MagLead system; Matsudo, Japan) (*n* = 1). For nucleic acid purification, the starting volume varied between laboratories (Table 3).

### Real-time RT-qPCR molecular detection methodology

The real-time RT-qPCR kits used by the laboratories are presented in Table 3. Five laboratories used the RT-qPCR one-step SuperScript III Platinum kit manufactured by Thermo Fisher Scientific, and two laboratories used the RT-qPCR kits from Meridian Bioscience (Cincinnati, OH, USA). The TaqMan RT-qPCR kit from Thermo Fischer Scientific and RT-qPCR kits from Promega and NZYtech (Lisbon, Portugal) were used by two laboratories of which laboratory #5 used both the Thermo Fisher Scientific TaqMan and NZYtech kits.

### Conventional RT-PCR molecular detection methodology

Table 3 presents the techniques used for conventional RT-PCR molecular detection methods. Six laboratories used three different Thermo Fisher Scientific kits. Kits from Meridian Bioscience (*n* = 1), New England Biolabs (Ipswich, MA, USA) (*n* = 1) and Qiagen (*n* = 1) were also used.

### Real-time RT-qPCR and conventional RT-PCR results

#### TOSV samples using Toscana virus trio assay

All nine laboratories successfully detected the two TOSV-positive samples. The Ct values varied for both TOSV lineage A (25.9–32.8) and for TOSV lineage B (23.3–33.5) (Table 4). None of the laboratories reported results showing problems of carry-over contamination.

**Table 4:** Results from the Toscana virus- and Sandfly fever Sicilian virus-specific reverse-transcriptase PCR assays and from the Pan-generic real-time PCR assays, from the different participating laboratories.

Laboratory identity number	TOSV (positive n=2)	TOSV (Lineage A and B), Ct	%^b^	Negative (n=6)	Pan-SFSV (positive n=1)	SFSV Ct^a^	%^b^	Negative (n=7)	Pan-Phlebovirus (positive n=6)	Detected phleboviruses^c^	%^b^	Negative (n=2)	Total^b^
Reference Laboratory	2	TOSV A 26.2 TOSV B 26.4	100	6	1	26.0	100	7	6	PUNV,ARBV, MASV, SFSV, TOSV A and B	100	2	100%
1	2	TOSV A 26.6 TOSV B 27.1	100	6	1	26.0	100	7	6	PUNV,ARBV, MASV, SFSV, TOSV A and B	100.0	2	100.0%
2	2	TOSV A 28.9 TOSV B 28.1	100	6	1	28.6	100	7	5	PUNV,ARBV, MASV, SFSV, TOSV A	83.3	3	83.3%
3	2	TOSV A 32.8 TOSV B 32.1	100	6	1	29.2	100	7	2	PUNV,ARBV,	33.3	6	77.7%
4	2	TOSV A 25.9 TOSV B 26.1	100	6	1	23.3	100	7	3	PUNV,ARBV, MASV	50.0	5	83.3%
5	2	TOSV A 30.0 TOSV B 30.0	100	6	1	31.0	100	7	5	PUNV,ARBV, MASV, SFSV, TOSV A	83.3	3	94.4%
6	2	TOSV A 31.8 TOSV B 33.4	100	6	1	28.8	100	7	5	PUNV,ARBV, MASV, SFSV, TOSV A	83.3	3	94.4%
7	2	TOSV A 31.2 TOSV B 33.5	100	6	1	29.7	100	7	3	PUNV,ARBV, MASV	50.0	5	83.3%
8	2	TOSV A 28.9 TOSV B 29.5	100	6	1	27.1	100	7	5	PUNV,ARBV, MASV, SFSV, TOSV A	83.3	3	94.4%
9	2	TOSV A 31.0 TOSV B 32.0	100	6	1	30.0	100	7	3	PUNV,ARBV, MASV	50.0	5	83.3%

*SFSV* Sandfly fever Sicilian virus. For definitions of ARBV, MASV, PUNV and TOSV, see Table 1

^a^The specific RT-qPCR assay outcomes are included as cycle threshold (Ct) values

^b^The accuracy percentage was calculated for each individual assay and for the combined results (Total column)

#### SFSV sample using the Pan-SFSV assay

All nine laboratories successfully detected the SFSV-positive sample, with Ct values ranging from 26.0 to 31.0 (Table 4). None of the laboratories reported results showing problems of carry-over contamination.

#### All samples using the Pan-Phlebovirus assay

Only laboratory #1 successfully detected all six samples of SbPV (Table 4). Four laboratories detected five out of six positive samples, three laboratories detected three positive samples and one laboratory detected two positive samples (Table 4). Punique virus (PUNV) and Arbia (ARBV) virus were detected by all nine laboratories, Massilia virus (MASV) was detected by eight laboratories, SFSV and TOSV lineage A was detected by 5 laboratories and TOSV lineage B was detected by only one laboratory.

### Evaluation of detection success rate

For the TOSV trio assay and Pan-SFSV assay, all laboratories had 100% success of detection. In contrast, sample detection ranged between 33.3% and 100% for the Pan-Phlebovirus assay, with one laboratory detecting all six (100%) positive samples, and four, three and one laboratories detecting five (83.3%), three (50%) and one (33.3%) samples, respectively (Table 4). The overall SbPV detection success of the EQA was between 77.7% and 100% (Table 4).

## Discussion

This EQA was organized within the framework of the EU CLIMOS Project to standardize detection of SbPV in order to provide comparable data to feed mathematical models for the surveillance of the impact of climate changes and environmental parameters on the kinetics and diversity of sand fly species and on the sand fly-borne microorganisms. Accordingly, the nine laboratories participating in this EQA were those participating in the CLIMOS project.

The distribution of sand flies in the Mediterranean region is well documented [13, 14]. Recent studies suggest that the relationship between SbPV and their phlebotomine vector species is not as restrictive as once thought [5]. It is likely that different SbPV can be transmitted by the same sand fly species and that a single sand fly species may carry different SbPV. In addition, sand flies are rarely tested individually but rather pooled in groups of 30 or 50 individual flies, so that the chance that two viruses are present in the same pool is not negligible. To better understand SbPV circulation, robust detection assays are essential, particularly those capable of being deployed across wide geographic regions.

Over the last few decades, numerous studies have sought to screen vector-borne SbPV, leading to the discovery of novel SbPV and the detection of known ones in previously unrecognized areas [1–3, 15–17]. The ability to detect and to identify circulating SbPV is also crucial for recognizing potential pathogens and enhancing prevention methods and strategies.

The three assays selected for testing in this study are intended for use in epidemiological studies and virus discovery/detection studies. They are not meant for diagnostic purposes, with the exception of the TOSV trio assay [12].

We targeted two known pathogens in this EQA, TOSV and SFSV. For TOSV, a specific trio RT-qPCR assay was selected, comprising three monoplex systems. This assay was chosen because it had been previously validated also for the clinical diagnostics of TOSV infection [12]. For SFSV, we selected a real-time RT-qPCR assay that allows detection of not only SFSV but also the genetically closely related CFUV, DASV and TORV identified in Greece, Iran and Türkiye, respectively [16]. Interestingly, CFUV, DASV and TORV are now considered to be unique members of the *Phlebovirus corfouense, Phlebovirus dashliense* and *Phlebovirus toroense* species respectively. Whether these related viruses are pathogenic to humans remains unknown. It should also be noted that SFSV is also the unique member of the *Phlebovirus siciliaense*.

Additionally, the single Pan-Phlebovirus RT-PCR assay was able to detect the novel and already recognized SbPV (such as ARBV, MASV and PUNV viruses) incorporated into the EQA. For selecting the pan-generic assay we relied on a study that compared four assays [4]; then, based on these findings, we selected the system developed by Matsuno et al. [10] as it demonstrated a good balance between sensitivity and detection spectrum.

Among the six vials containing phleboviruses, the two TOSV and one SFSV samples were detectable not only using the RT-qPCR primers and probes but also by the conventional and RT-PCR reagents. The contents of three vials were detectable by conventional RT-PCR reagents only, namely MASV, PUNV and ARBV. The two latter viruses were detected by all nine participating laboratories whereas MASV was detected by eight of the nine laboratories. It should be emphasized here that species identification was not part of the EQA trial since it requires additional sequencing techniques.

Although all laboratories successfully identified the two TOSV and the one SFSV using the proposed RT-qPCR assays, there was some variations in the Ct values across laboratories, possibly due to differences in RNA extraction and/or RT-qPCR sensitivity. Four laboratories using manual extraction kits reported higher Ct values, potentially indicating RNA loss during extraction, which is consistent with previous findings that manual extraction can result in lower viral RNA yield compared to automated protocols [18]. For the Pan-Phlebovirus RT-PCR assay, the SbPV detection rate was lower than that of the specific real-time RT-qPCR assays. As the results of the specific real-time RT-qPCR assays showed, the laboratories who had high Ct values for the TOSV and SFSV samples were unable to detect these samples with the Pan-Phlebovirus RT-PCR assay. This outcome reflects the known trade-off between the broad detection spectrum and reduced sensitivity of pan-generic PCR assays. As expected, the RT-qPCR assays showed a better sensitivity for detecting TOSV and SFSV compared to the Pan-Phlebovirus generic RT-PCR assay, as previously shown for TOSV [19]. The objectives of the two types of tests were not the same: (i) the specific real-time TOSV test is part of a detection/diagnosis logic, whereas (ii) the pan-generic test approach (Pan-SFSV and Pan-Phlebovirus) corresponds to a more open approach aimed at discovering new viruses and assessing the presence of recognized viruses in a given geographic area. The overall success rate for SbPV detection ranged from 77.7% to 100%, demonstrating the high detection capability among participating laboratories.

It can be argued that strictly speaking, the study reported here is not an EQA since the molecular detection tools were distributed to the participating laboratories. Laboratories provided their own nucleic acid extraction techniques, reverse transcriptase and Mastermix kits and PCR equipment. Moreover, most of the participating laboratories did not routinely carry out molecular detection of SbPV prior to this project, except for the Italian and Israeli laboratories. The observed, satisfactory results show that providing freeze-dried reagents for diagnosis allows accurate detection within a timeframe compatible with the completion of a scientific project. Indeed, these laboratories are now implementing phlebovirus detection locally on trapped sand flies without the need to ship material to a reference laboratory. Our results also lay the groundwork for the rapid deployment of a detection or diagnostic capability on a site that has the technical tools, in the absence of specific expertise in the field.

The aim of this EQA was not only to demonstrate the laboratories' abilities to detect SbPV, but also to improve the use of molecular tools that will enable research institutions to set up or take part in research and surveillance programs in the fields of "virus discovery" or "virus detection" in addition to their primary areas of expertise. By strengthening infrastructure and expertise, capacity-building initiatives improve the ability of institutions to conduct accurate and timely molecular diagnostics, to contribute to research and development and to respond effectively to emerging public health challenges. Collaboration and knowledge-sharing among international laboratories are crucial components of these efforts. Ultimately, molecular capacity-building empowers countries to address local health issues more effectively and contributes to global health security.

## Conclusions

This EQA, conducted within the framework of the EU CLIMOS Project, successfully harmonized the molecular detection of SbPV by nine laboratories, enabling comparable data to be obtained for climate and environmental impact modeling. The study confirmed the high sensitivity of specific RT-qPCR assays for TOSV and SFSV, while the Pan-Phlebovirus RT-PCR assay proved valuable for broader virus detection despite its lower sensitivity.

Variation in Ct values, particularly higher Ct values from laboratories with using manual RNA extraction, highlights probable RNA loss during extraction. Importantly, this EQA facilitated the implementation of molecular detection tools in previously inexperienced laboratories, demonstrating the feasibility of decentralized viral screening.

Beyond evaluating detection capabilities, this EQA aimed to strengthen molecular capacity, supporting virus surveillance and discovery efforts. By fostering international collaboration, it enhances preparedness for emerging infectious diseases and contributes to global health security.

## Supplementary Information

**Additional file 1: Data file 1.**

**Additional file 2: Data file 2.**

## Data Availability

Data is provided within the manuscript or supplementary information files.

## Abbreviations

ARBV: Arbia virus
CFUV: Corfou virus
DASV: Dashli virus
EQA: External Quality Assessment
EU: European Union
F: Forward
L: Large segment
Lyo-P&P: Lyophilized primers and probes
MASV: Massilia virus
N: Viral nucleocapsid protein
Nts: Nucleotides
P: Probe
PUNV: Punique virus
PCR: Polymerase chain reaction
R: Reverse
RT-qPCR: Reverse-transcriptase PCR
S: Small segment
SbPV: Sand fly-borne phleboviruses
SFSV: Sandfly fever Sicilian virus
SOP: Standard operating procedure
TORV: Toros virus
TOSV: Toscana virus
