# Supplementary material for: External quality assessment for molecular detection of sand fly-borne phleboviruses circulating in the Mediterranean Basin
Source: Parasit Vectors. 2025 May 12;18:173. doi: 10.1186/s13071-025-06785-0 (PMC12070498; doi:10.1186/s13071-025-06785-0)
Supplement: Supplementary file 1 — Additional file 1: Data file 1. [file 13071_2025_6785_MOESM1_ESM.pdf]

STANDARD OPERATING PROCEDURE

Phlebovirus External Quality Assessment (EQA) Protocol

Nº SOP	SOP CLIMOS# 3.1
Date	Version
Elaborated by	Nazli Ayhan, Remi Charrel, Gioia Bongiorno
Approved by	Carla Maia

PURPOSE

The purpose of this Standard Operating Procedure (SOP) is to describe the protocol used for External Quality Assessment (EQA)

INTRODUCTION

The term external quality assessment (EQA) is used to describe a method that allows comparison of a laboratory's testing to an external source. The performance of a peer group of laboratories or the performance of a reference laboratory can be compared [1].

This EQA contains;

- 8 vials of inactivated or mock samples to be tested
- 3 vials of lyophilized primers and probes (lyoph-P&P)

These 11 vials should be stored at -20°C upon reception before being processed.

UK Research and Innovation

UK Research and Innovation, Horizon Europe Guarantee.

Co-funded by the European Union

Funded by the European Union. Views and opinions expressed are however those of the author(s) only and do not necessarily reflect those of the European Union or the European Health and Digital Executive Agency (HADEA). Neither the European Union nor the granting authority can be held responsible for them.

MATERIALS NEEDED

Samples

- 8 vials of inactivated or mock samples to be tested
- 3 vials of lyophilized primers and probes (lyoph-P&P)

Buffers and Solutions

- Sterile distilled water or molecular grade water

Equipment and Consumables

- Freezer (- 20°C)
- Thermal cycler
- Real-time thermal cycler
- Agarose gel electrophoresis and visualization system
- 1.5 ml Eppendorf tubes
- 0.2 ml PCR tubes and/or 96 well PCR plates
- Pencils, permanent pen
- Eppendorf and PCR tube storage boxes
- Micropipettes (0.1 – 1000 µl) and pipette tips
- Centrifuge
- Ice blocks / cold racks
- Commercial DNA extraction kits and commercial RNA extraction kits or commercial nucleic acid extraction kits
- PCR reagents (Taq polymerase, PCR master mix, primers, probes, molecular-grade water)
- Agarose, nucleic acid stain, nucleic acid loading dye, DNA ladder

METHOD

M1. Sample Processing

- Freeze-dried material, to be stored at -20°C upon reception before being processed.

M2. Resuspension of EQA vials

All 8 vials must be reconstituted as indicated below. For each vial;

- Add **400µL** of sterile distilled water.

UK Research and Innovation
Co-funded by the European Union

UK Research and Innovation, Horizon Europe Guarantee.

Funded by the European Union. Views and opinions expressed are however those of the author(s) only and do not necessarily reflect those of the European Union or the European Health and Digital Executive Agency (HADEA). Neither the European Union nor the granting authority can be held responsible for them.

- Homogenize by pipetting: adjust the pipette 200µl and pipet 10 times.
- Incubation 10 minutes on the bench (room temperature).

M3. Nucleic Acid Extraction

- Depending on the extraction protocol chosen by the partners, the extracted RNA or total nucleic acid, should be eluted in a volume ranging from 60 to100uL.

M4. Resuspension of Lyophilized Primers and Probes (Lyoph-P&P)

Each tube contains lyoph-P&P for 24 reactions for three different detection systems: TOSV [2], SFSV [3] and PanPhlebo [4]. All 3 vials must be reconstituted as indicated below. For each vial;

- Add 182µl sterile distilled water.
- Homogenize by pipetting: adjust the pipette 100µl and pipet 10 times.
- Vortex the samples for about 5 seconds.
- Incubate ≥10 minutes on the bench (room temperature) before use (VERY IMPORTANT).

Attention: Lyoph-P&P should be resuspended immediately before PCR.

UK Research and Innovation

Co-funded by the European Union

UK Research and Innovation, Horizon Europe Guarantee.

Funded by the European Union. Views and opinions expressed are however those of the author(s) only and do not necessarily reflect those of the European Union or the European Health and Digital Executive Agency (HADEA). Neither the European Union nor the granting authority can be held responsible for them.

M5. Conventional RT-PCR for Pan-Phlebovirus (Matsuno)

- Kit Recommendation: SuperScript™ One-Step RT-PCR System with Platinum (reference number: Thermo fisher 10928042)

Reagent	Volume	Volume 10x
Buffer (µL)	25	250
Primers lyophilize (µL)<br>Pan-Phlebo (Matsuno)	4	40
Enzyme SSIII Platinium (µL)	1	10
H_2O (µL)	15	150

- If another kit is used, please adapt the volumes to the manufacturer's recommendations.

Cycling

50°C	30 min	
94°C	2 min	
94°C	30 sec	40 cycles
55°C	1.5 min	
68°C	30 sec	
68°C	7 min	
20°C	2 min	

Interpretation of results

- Products of RT-PCR amplification will be evaluated by electrophoresis in a 2.0% agarose gel.

  The observation of a ~500 nt band a priori reveals the presence of a phlebovirus genome.

  Please note that it is possible to have double bands for some phlebovirus strains (~500 nt and ~550nt).

UK Research and Innovation

Co-funded by the European Union

UK Research and Innovation, Horizon Europe Guarantee.

Funded by the European Union. Views and opinions expressed are however those of the author(s) only and do not necessarily reflect those of the European Union or the European Health and Digital Executive Agency (HADEA). Neither the European Union nor the granting authority can be held responsible for them.

M6. Real-time RT-qPCR **Molecular Consensus Assay Amplification of** TOSV and SFSV

- Kit Recommendation: One-step qRT-PCR one-step Superscript III Platinum (reference number Thermo fisher 11732088) (for 500 reactions).

Reagent	Volume	Volume 10x
Mix 2X PCR (µL)	12.5	125
Enzyme RT (µL)	0.5	5
P&P lyophilize (µL) (TOSV or SFSV primers)	7	70
Volume total (µL)	20	200

- If another kit is used, please adapt the volumes to manufacturer's recommendations.

Cycling

50°C	15 min	
95°C	2 min	
95°C	15 sec	45 cycles
60°C	45 sec	

Interpretation of results

- The positive sample should have an amplification curve with a cycle threshold value (Ct).

Submission of results

Please send your results in an excel file provided by the/a reference laboratory.

You will need the following information to submit your results:

• Date receipt EQA panel and date analysis panel (if multiple days, date of RNA extraction)

• Storage conditions of the samples and condition of the panel (upon arrival)

• Detailed information on RNA or nucleic acid extraction process (incl. automated or manual process, exact name of the extraction system and/or the extraction kit)

• Reconstitution volume for samples of the panel, input volume used for RNA extraction, elution volume used for RNA extraction

• Detailed information on PCR method (incl. PCR kit name)

• Ct values and presence/absence of a gel band in targeted region for all samples.

UK Research and Innovation

Co-funded by the European Union

UK Research and Innovation, Horizon Europe Guarantee.

Funded by the European Union. Views and opinions expressed are however those of the author(s) only and do not necessarily reflect those of the European Union or the European Health and Digital Executive Agency (HADEA). Neither the European Union nor the granting authority can be held responsible for them.

Contact

Please refer for additional information to the technical document provided with the panel or contact Nazli AYHAN, Ph.D (nazliayhann@gmail.com).

REFERENCES

1. Thirion L, et al. Lyophilized Matrix Containing Ready-to-Use Primers and Probe Solution for Standardization of Real-Time PCR and RT-qPCR Diagnostics in Virology. Viruses. 2020 Jan 30;12(2). pii: E159. doi: 10.3390/v12020159. PubMed PMID: 32019076.
2. Thirion, L., Pezzi, L., Pedrosa-Corral, I., Sanbonmatsu-Gamez, S., De Lamballerie, X., Falchi, A., Perez-Ruiz, M. and Charrel, R.N., 2021. Evaluation of a Trio Toscana Virus Real-Time RT-PCR Assay Targeting Three Genomic Regions within Nucleoprotein Gene. Pathogens, 10(3), p.254.
3. Alwassouf, S., Maia, C., Ayhan, N., Coimbra, M., Cristovao, J.M., Richet, H., Bichaud, L., Campino, L. and Charrel, R.N., 2016. Neutralization-based seroprevalence of Toscana virus and sandfly fever Sicilian virus in dogs and cats from Portugal. Journal of General Virology, 97(11), pp.2816-2823.
4. Matsuno, K., Weisend, C., Kajihara, M., Matysiak, C., Williamson, B.N., Simuunza, M., Mweene, A.S., Takada, A., Tesh, R.B. and Ebihara, H., 2015. Comprehensive molecular detection of tick-borne phleboviruses leads to the retrospective identification of taxonomically unassigned bunyaviruses and the discovery of a novel member of the genus phlebovirus. Journal of virology, 89(1), pp.594-604.

DOCUMENT EDITING HISTORY

Version Nº	Elaborated by	Revised by	Approved by	Editing date	Summary of changes
0.1	<Name>	<Name>	<Name>	Yyyy/mm/dd	<Text>

UK Research and Innovation

Co-funded by the European Union

UK Research and Innovation, Horizon Europe Guarantee.

Funded by the European Union. Views and opinions expressed are however those of the author(s) only and do not necessarily reflect those of the European Union or the European Health and Digital Executive Agency (HADEA). Neither the European Union nor the granting authority can be held responsible for them.
